# Supplementary figures and images for: ExhauFS: exhaustive search-based feature selection for classification and survival regression
Source: PeerJ. 2022 Mar 30;10:e13200. doi: 10.7717/peerj.13200 (PMC8976470; doi:10.7717/peerj.13200)

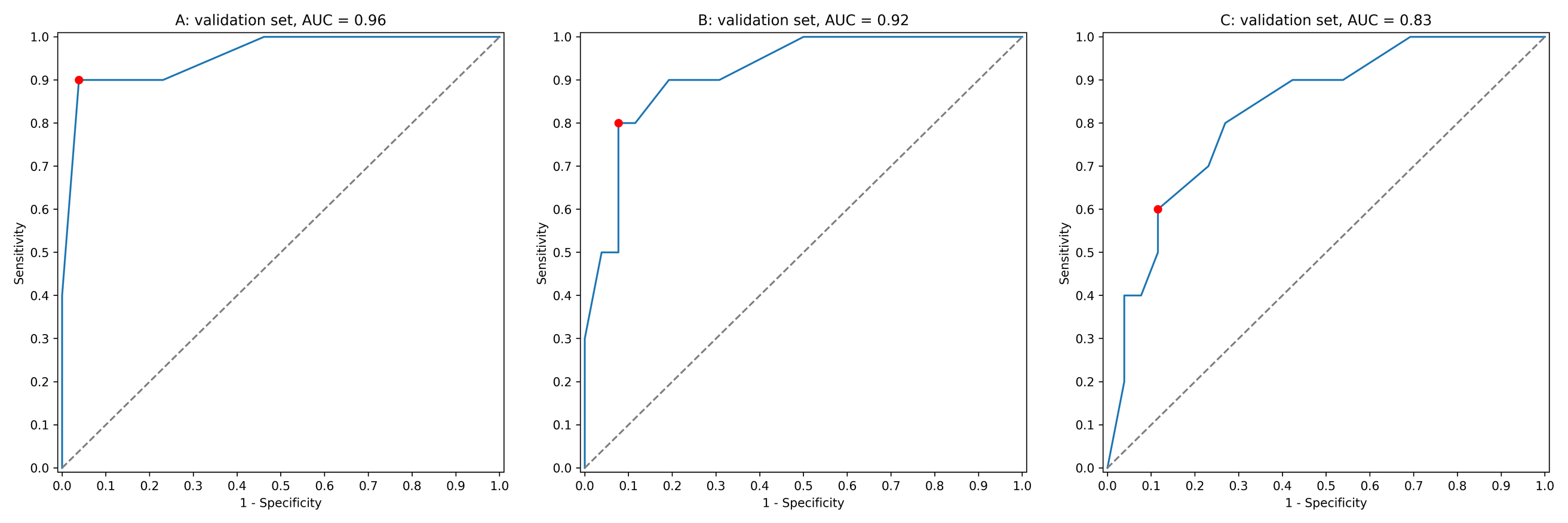

Supplement: Figure S1 — (A) Three best features found by ExhauFS (“perception_vulnerability” “socialSupport_instrumental”, “empowerment_desires”). (B) Random forest classifier applied to the whole set of features. (C) Three most important features found by random forest classifier (“behavior_personalHygine”, “empowerment_knowledge”, “perception_severity”). Red points on ROC curves stand for the actual random forest classifier threshold values. [file peerj-10-13200-s001.png]

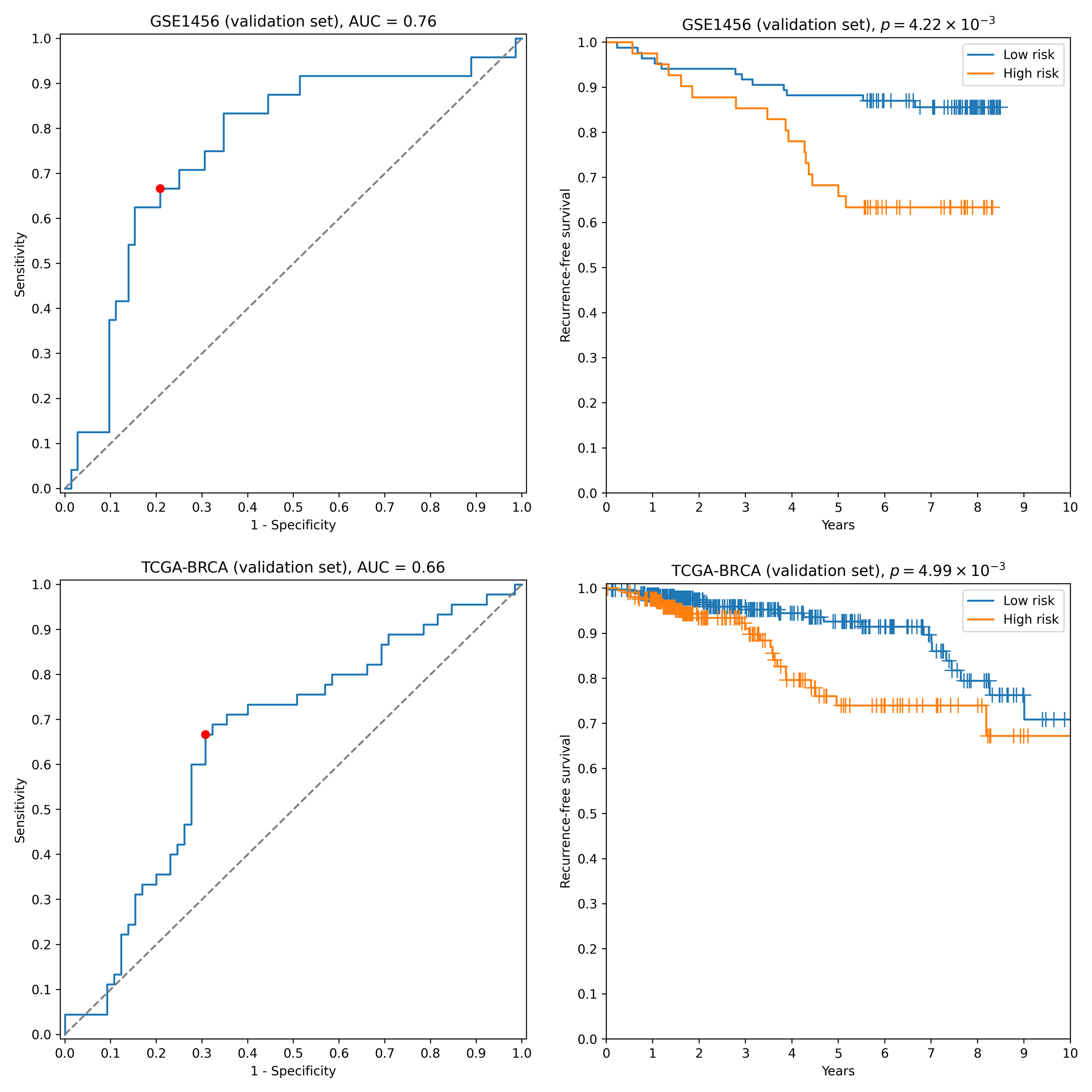

Supplement: Figure S2 — Red points on ROC curves stand for the actual SVM threshold values. [file peerj-10-13200-s002.png]

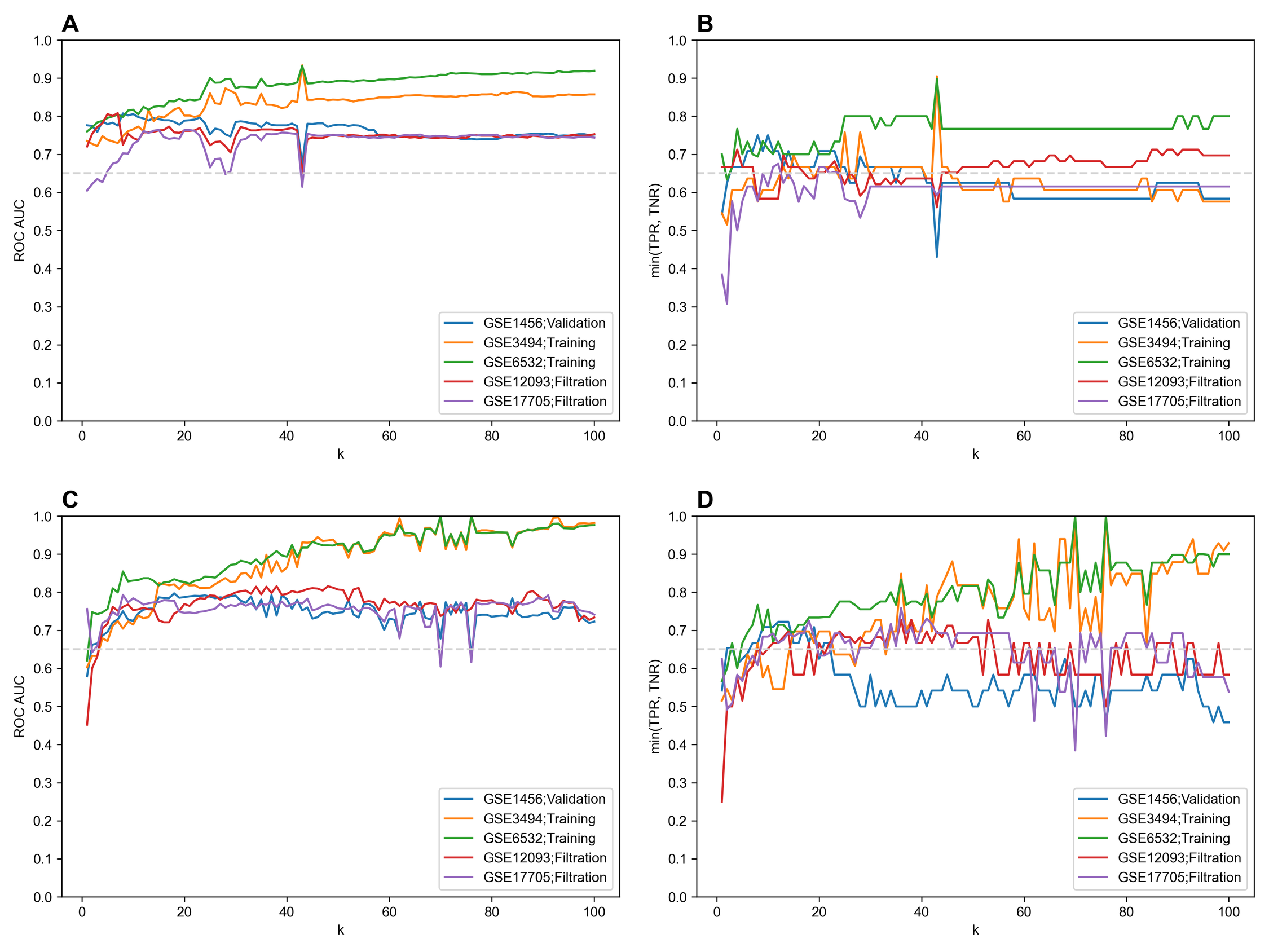

Supplement: Figure S3 — (A, B) Univariate feature selection (t-test). (C, D) L1-regularized logistic regression. [file peerj-10-13200-s003.png]

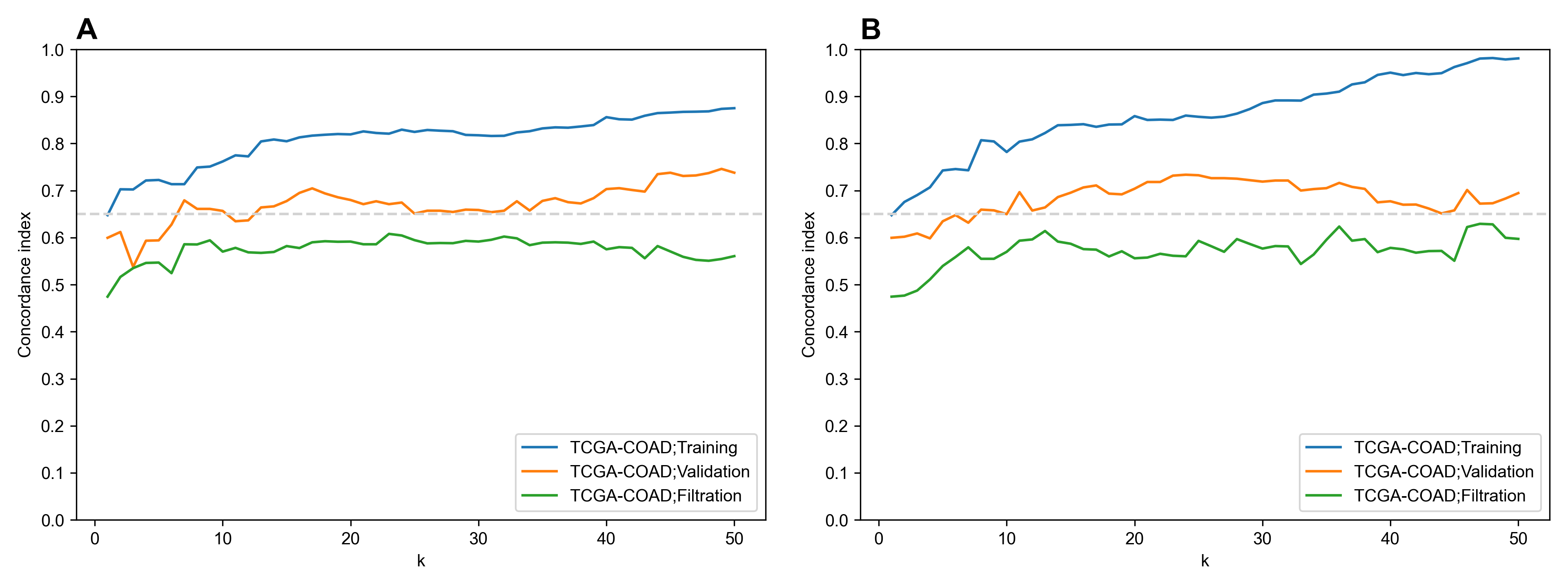

Supplement: Figure S4 — (A) Univariate feature selection (concordance index). (B) L1-regularized Cox model. [file peerj-10-13200-s004.png]
