# Supplementary material for: ExhauFS: exhaustive search-based feature selection for classification and survival regression
Source: PeerJ. 2022 Mar 30;10:e13200. doi: 10.7717/peerj.13200 (PMC8976470; doi:10.7717/peerj.13200)
Supplement: Supplemental Information 5 [file peerj-10-13200-s005.docx]

**Table S1. Numbers of patients in breast cancer datasets.**

| **Dataset** | **Number of patients with recurrence during the first 5 years** | **Number of recurrence-free patients with at least 7 years follow-up** | **Total number of patients (including the grey 5-7 years zone)** |
| --- | --- | --- | --- |
| GSE1456 | 24 | 72 | 126 |
| GSE3494 | 33 | 42 | 202 |
| GSE6532 | 30 | 49 | 151 |
| GSE12093 | 12 | 66 | 135 |
| GSE17705 | 26 | 120 | 189 |
| TCGA-BRCA | 45 | 66 | 559 |

**Table S2. Performance of the individual feature-based models for the toy example***.*

|  | **Validation set** | | | **Training set** | | |
| --- | --- | --- | --- | --- | --- | --- |
| **Feature** | **TPR** | **TNR** | **ROC AUC** | **TPR** | **TNR** | **ROC AUC** |
| empowerment_abilities | 0.5 | 0.692 | 0.56 | 0.909 | 0.88 | 0.958 |
| socialSupport_emotionality | 0.7 | 0.692 | 0.798 | 0.909 | 0.8 | 0.904 |
| perception_severity | 0.7 | 0.577 | 0.733 | 0.909 | 0.8 | 0.904 |
| empowerment_knowledge | 0.7 | 0.769 | 0.771 | 0.727 | 0.88 | 0.904 |
| norm_fulfillment | 0.6 | 0.731 | 0.767 | 0.727 | 0.88 | 0.904 |
| intention_aggregation | 0.2 | 0.731 | 0.588 | 0.727 | 0.88 | 0.873 |
| socialSupport_appreciation | 0.7 | 0.692 | 0.654 | 0.727 | 0.8 | 0.844 |
| norm_significantPerson | 0.6 | 0.577 | 0.558 | 0.727 | 0.76 | 0.765 |
| behavior_eating | 0.7 | 0.5 | 0.64 | 0.727 | 0.64 | 0.72 |
| motivation_willingness | 0.7 | 0.923 | 0.804 | 0.636 | 0.96 | 0.92 |
| perception_vulnerability | 0.5 | 0.731 | 0.754 | 0.636 | 0.92 | 0.92 |
| behavior_personalHygine | 0.3 | 0.885 | 0.694 | 0.636 | 0.92 | 0.898 |
| attitude_consistency | 0.5 | 0.577 | 0.488 | 0.545 | 0.8 | 0.76 |
| empowerment_desires | 0.3 | 0.962 | 0.483 | 0.455 | 1.0 | 0.856 |
| motivation_strength | 0.5 | 0.846 | 0.719 | 0.455 | 1.0 | 0.769 |
| socialSupport_instrumental | 0.4 | 0.923 | 0.779 | 0.455 | 0.96 | 0.796 |
| intention_commitment | 0.2 | 1.0 | 0.66 | 0.273 | 0.96 | 0.707 |
| behavior_sexualRisk | 0.4 | 0.923 | 0.662 | 0.182 | 1.0 | 0.591 |
| attitude_spontaneity | 0.0 | 1.0 | 0.335 | 0.0 | 1.0 | 0.651 |

**Table S3. Summary table for breast cancer prognostic classifiers constructed without feature pre-selection.** *N* filtration stands for number of classifiers which passed the filtration step, *N* validation stands for number of classifiers which additionally passed the same accuracy thresholds on the validation set.

| ***n*** | ***k*** | ***N* filtration** | ***N* validation** | **Percentage (validation / filtration)** |
| --- | --- | --- | --- | --- |
| 7762 | 1 | 0 | 0 | 0% |
| 1000 | 2 | 3 | 1 | 33.3% |
| 130 | 3 | 65 | 35 | 53.8% |
| 50 | 4 | 10 | 3 | 30% |
| 32 | 5 | 17 | 5 | 29.4% |
| 24 | 6 | 0 | 0 | 0% |
| 21 | 7 | 0 | 0 | 0% |
| 19 | 8 | 0 | 0 | 0% |
| 18 | 9 | 0 | 0 | 0% |
| 18 | 10 | 0 | 0 | 0% |
| 19 | 11 | 0 | 0 | 0% |
| 19 | 12 | 0 | 0 | 0% |
| 19 | 13 | 0 | 0 | 0% |
| 20 | 14 | 0 | 0 | 0% |
| 20 | 15 | 0 | 0 | 0% |
| 21 | 16 | 0 | 0 | 0% |
| 22 | 17 | 0 | 0 | 0% |
| 23 | 18 | 0 | 0 | 0% |
| 23 | 19 | 0 | 0 | 0% |
| 24 | 20 | 0 | 0 | 0% |

The last column of the table (total number of patients) stands for datasets used in Kaplan-Meier plots construction (Figure 2, Figure S2).

**Table S4. Summary table for breast cancer prognostic classifiers constructed with “stable” genes pre-selection.**

| ***n*** | ***k*** | ***N* filtration** | ***N* validation** | **Percentage (validation / filtration)** |
| --- | --- | --- | --- | --- |
| 7762 | 1 | 0 | 0 | 0% |
| 1000 | 2 | 0 | 0 | 0% |
| 130 | 3 | 15 | 4 | 26.7% |
| 50 | 4 | 265 | 90 | 34.0% |
| 32 | 5 | 1706 | 534 | 31.3% |
| 24 | 6 | 836 | 532 | 63.6% |
| 21 | 7 | 1410 | 1049 | 74.4% |
| 19 | 8 | 1686 | 1433 | 85.0% |
| 18 | 9 | 1498 | 1381 | 92.2% |
| 18 | 10 | 1773 | 1696 | 95.7% |
| 19 | 11 | 3607 | 3515 | 97.4% |
| 19 | 12 | 2990 | 2935 | 98.2% |
| 19 | 13 | 1879 | 1860 | 99.0% |
| 20 | 14 | 2779 | 2750 | 98.9% |
| 20 | 15 | 1272 | 1261 | 99.1% |
| 21 | 16 | 1343 | 1335 | 99.4% |
| 22 | 17 | 1672 | 1663 | 99.5% |
| 23 | 18 | 3470 | 3401 | 98.0% |
| 23 | 19 | 1156 | 1144 | 99.0% |
| 24 | 20 | 549 | 539 | 98.2% |

*N* filtration stands for number of classifiers which passed the filtration step, *N* validation stands for number of classifiers which additionally passed the same accuracy thresholds on the validation set.

**Table S5. Number of gene occurrences in signatures which passed the filtration step**.

| **Gene** | **Count** | **Percentage**  **(count / 1773)** | ***p*-value** | **Adjusted *p*-value** |
| --- | --- | --- | --- | --- |
| *ABAT* | 1666 | 94.0% | 1.20E-290 | **2.15E-289** |
| *CCNL2* | 1356 | 76.5% | 1.42E-75 | **1.28E-74** |
| *TRIP13* | 1256 | 70.8% | 2.48E-40 | **1.49E-39** |
| *EPN3* | 1251 | 70.6% | 6.83E-39 | **3.07E-38** |
| *IGFBP6* | 1158 | 65.3% | 2.61E-17 | **9.41E-17** |
| *ZWINT* | 1071 | 60.4% | 1.65E-05 | **4.95E-05** |
| *CX3CR1* | 1025 | 57.8% | 0.0262901 | 0.06760311 |
| *MB* | 1015 | 57.2% | 0.07231083 | 0.16269938 |
| *SLC7A5* | 946 | 53.3% | 0.96698141 | 1 |
| *ECHDC2* | 929 | 52.4% | 0.99595152 | 1 |
| *UBE2C* | 893 | 50.4% | 0.99999352 | 1 |
| *KIF4A* | 850 | 48.0% | 1 | 1 |
| *NUMA1* | 836 | 47.1% | 1 | 1 |
| *CFAP69* | 822 | 46.4% | 1 | 1 |
| *MTFR1* | 817 | 46.1% | 1 | 1 |
| *RTN1* | 799 | 45.1% | 1 | 1 |
| *STARD13* | 646 | 36.4354202 | 1 | 1 |
| *GINS2* | 394 | 22.2222222 | 1 | 1 |

Data is shown for n = 18, k = 10. Total number of considered gene signatures: 1773. Binomial test (1773 trials with success probability 10/18) was used to calculate *p*-values, multiple testing correction was done using Benjamini-Hochberg procedure.

**Table S6. Summary table for colorectal cancer prognostic regressors (concordance index-based feature selection).**

| ***n*** | ***k*** | ***N* filtration** | ***N* validation** | **Percentage (validation / filtration)** |
| --- | --- | --- | --- | --- |
| 86 | 1 |  | 0 | 0% |
| 86 | 2 |  | 0 | 0% |
| 86 | 3 |  | 0 | 0% |
| 59 | 4 | 332 | 27 | 8.1% |
| 37 | 5 | 1699 | 306 | 18.0% |
| 28 | 6 | 2954 | 720 | 24.4% |
| 24 | 7 | 7493 | 2435 | 32.5% |
| 22 | 8 | 8979 | 3874 | 43.1% |
| 21 | 9 | 8488 | 4558 | 53.7% |
| 20 | 10 | 5040 | 3413 | 67.7% |
| 20 | 11 | 3905 | 2988 | 76.5% |
| 20 | 12 | 2358 | 1966 | 83.4% |
| 20 | 13 | 1042 | 923 | 88.6% |
| 21 | 14 | 1072 | 937 | 87.4% |
| 22 | 15 | 1467 | 1247 | 85.0% |
| 22 | 16 | 383 | 341 | 89.0% |
| 23 | 17 | 773 | 711 | 92.0% |
| 23 | 18 | 134 | 129 | 96.3% |
| 24 | 19 | 150 | 135 | 90.0% |
| 25 | 20 | 67 | 60 | 89.6% |

*N* filtration stands for number of Cox models which passed the filtration step, *N* validation stands for number of models which additionally passed the same accuracy thresholds on the validation set.

**Table S7. Summary table for colorectal cancer prognostic regressors (feature selection based on the search of differentially expressed isomiRs between normal tissues and tumors).**

| ***n*** | ***k*** | ***N* filtration** | ***N* validation** | **Percentage (validation / filtration)** |
| --- | --- | --- | --- | --- |
| 86 | 1 | 0 | 0 | 0% |
| 86 | 2 | 0 | 0 | 0% |
| 86 | 3 | 191 | 42 | 22.0% |
| 59 | 4 | 1504 | 534 | 35.5% |
| 37 | 5 | 5292 | 1605 | 30.3% |
| 28 | 6 | 4441 | 1 | 0.0% |
| 24 | 7 | 11 | 7 | 63.6% |
| 22 | 8 | 70 | 50 | 71.4% |
| 21 | 9 | 113 | 94 | 83.2% |
| 20 | 10 | 105 | 96 | 91.4% |
| 20 | 11 | 87 | 82 | 94.2% |
| 20 | 12 | 47 | 42 | 89.4% |
| 20 | 13 | 23 | 20 | 87.0% |
| 21 | 14 | 5 | 4 | 80.0% |
| 22 | 15 | 20 | 18 | 90.0% |
| 22 | 16 | 4 | 4 | 100.0% |
| 23 | 17 | 1 | 1 | 100.0% |
| 23 | 18 | 0 | 0 | 0% |
| 24 | 19 | 0 | 0 | 0% |
| 25 | 20 | 120 | 15 | 12.5% |

*N* filtration stands for number of Cox models which passed the filtration step, *N* validation stands for number of models which additionally passed the same accuracy thresholds on the validation set.
